# Delving into the Biotransformation Characteristics and Mechanism of Steamed Green Tea Fermented by *Aspergillus niger* PW-2 Based on Metabolomic and Proteomic Approaches

**DOI:** 10.3390/foods11060865

**Published:** 2022-03-18

**Authors:** Maoyun Li, Yue Xiao, Kai Zhong, Yanping Wu, Hong Gao

**Affiliations:** 1 College of Biomass Science and Engineering, Sichuan University, Chengdu 610065, China; myleecheer@163.com (M.L.); eric211@163.com (K.Z.); gao523@hotmail.com (H.G.); 2 West China School of Public Health, Sichuan University, Chengdu 610065, China; scu_xyue@163.com

**Keywords:** fermentation, tea quality, flavor, taste, metabolism, dark tea, chemical components

## Abstract

*Aspergillus niger* is one of the dominant microorganisms presented in dark tea fermentation. In this study, the biotransformation of steamed green tea leaves fermented by *A. niger* PW-2 was characterized using metabolomic and proteomic approaches. We observed that, after fermentation, the contents of volatile compounds contributing to the “green” aroma, including linalool, *L*-*α*-terpineol and geraniol, decreased significantly. Meanwhile, the astringency taste and contents of metabolites contributing to the taste (catechins) reduced significantly during fermentation. Additionally, the contents of theabrownins, which have health benefits, obviously increased. The bitter and umami tastes were also changed due to the variations in bitter-taste and umami-taste amino acids. We also found that glycoside hydrolases, tannases, catechol oxidases, peroxidases and laccases secreted by *A. niger* PW-2 were responsible for the metabolism of phenolic compounds and their derivatives (theaflavins, thearubingins and theabrownins). Finally, the metabolic pathways involved in the biosynthesis and degradation of characteristic metabolites were found to reveal the biotransformation characteristics of dark tea fermented with *A. niger* PW-2.

## 1. Introduction

Dark tea, a type of post-fermentation tea that has increasingly become a popular and a preferred choice by consumers, is produced by microbial fermentation. Dark tea has distinct sensory characteristics, such as a unique stale, fungal flavor and a mellow, smooth taste; it also has weak astringency [1]. Furthermore, dark tea has multiple health benefits that are associated with its potent antioxidant, hepatoprotective, hyperglycemic and hyperlipidemic activities [2].

The microbial fermentation process is considered the main factor responsible for the formation of color, aroma and taste characteristics of dark tea. Catechins, which have an astringent taste, are rich in green teas and are oxidized during microbial fermentation. They contribute to the increase in pigments that cause a reddish-brown color of tea infusions. Theabrownins, the most abundant pigments in dark tea, can alter the gut microbiota and reduce hepatic cholesterol; they can also decrease lipogenesis by inhibiting the intestinal FXR-FGF15 signaling pathway [3]. Theabrownins have also been demonstrated to exhibit a strong umami taste [4]. Additionally, free amino acids also play a vital role in the taste of tea. The levels of most free amino acids are significantly reduced after fermentation [4,5]. Aroma is also one of the most important criteria determining the quality and flavor of dark tea. The quantitative sensory description has been found to change from a “green” attribute to “fungal flower”, “flower”, “mint” and “woody” attributes during the manufacturing process of Fuzhuan brick tea [6]. The aromas of different types of dark teas can vary depending on the types of raw leaves and the species of microorganism [7].

*Aspergillus niger*, an important fungal species in biotechnology, is often used to produce various extracellular enzymes, such as amylase, aminopeptidase, pectinase, catalase, cellulase, esterase and lipase [8]. During the microbial fermentation process, *A. niger* is one of the predominant microbes in dark tea that contribute to the formation of the unique quality of dark tea [2]. Specifically, *A. niger* can produce high contents of theabrownins in dark tea and enhance the contents of volatiles including linalool oxides, β-ionone, geranial and 9,12-octadecadienoic acid [7,9]. The composition of flavonoids, glycerophospholipids, organo-oxygen compounds and fatty acids could also be altered during *A. niger* fermentation. Meanwhile, enzymes produced by *A. niger* could change the concentrations and compositions of metabolites in tea leaves during fermentation [10].

Dark tea is usually prepared from old, coarse and rough leaves of *Camellia sinensis* var. *sinensis* or *Camellia sinensis* var. *assamica*, and the enzyme deactivation is conducted by pan-fired treatment [11,12]. Steamed green tea with a needle shape is produced in Meitan County of Guizhou Province, China, using leaves of *Camellia sinensis* cv. *Qianmei* 601; the enzyme deactivation is conducted by steam treatment. Steamed green tea usually has a typical green note, which is distinct from the roasted taste of pan-fired green tea [13]. The typical green note generated as a result of the steam treatment may endow fermented tea with its characteristic flavor. However, research on the development of fermented steamed green tea is scarce. Therefore, *A. niger* PW-2 was applied to produce of a novel, unique fermented loose tea using Guizhou steamed green tea as a raw material. The characteristic metabolites such as volatiles, catechins, pigments, amino acids and alkaloids, which are responsible for the flavor and taste of tea, were determined to reveal how the tea quality is improved by *A. niger* PW-2 fermentation. To elucidate the mechanism by which fermentation affected the conversion of metabolites, proteomics technology was used to analyze enzymes produced by *A. niger* PW-2 during different fermentation stages. The enzymes detected were functionally annotated according to the Gene Ontology (GO), Kyoto Encyclopedia of Genes and Genomes (KEGG) and Carbohydrate-Active enZYmes (CAZy) databases.

## 2. Materials and Methods

### 2.1. Materials

Pingwu Fuzhuan brick tea was obtained from Pingwu Xuebaoding Cha Industry Development Co., Ltd. (Sichuan, China). Steamed green tea leaves were obtained from Guizhou Gengtian Modern Agriculture Investment and Development Co. Ltd. (Guizhou, China). Potato dextrose agar (PDA), czapek dox agar (CDA), czapek yeast agar (CYA) and malt extract agar (MEA) were purchased from Hangzhou Microbial Reagent Co., Ltd. (Zhejiang, China). Caffeine, theobromine, theophylline, gallic acid (GA), epicatechin (EC), epigallocatechin gallate (EGCG), epicatechin gallate (ECG), epigallocatechin (EGC), catechin (C) and an *n*-alkane mixture (C_6_–C_24_) were purchased from Sigma-Aldrich Co. (St. Louis, MO, USA). A standard solution of amino acids was purchased from MembraPure GmbH Co. (Berlin, Germany). All other chemicals and reagents were purchased from Chengdu Kelong Chemical Reagent Factory (Sichuan, China).

### 2.2. Isolation and Identification of A. niger

**Figure 1:**
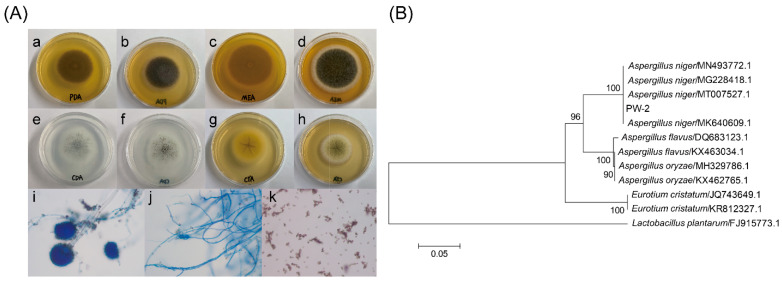
The morphological and molecular identification of *Aspergillus niger* PW-2 isolated from Pingwu Fuzhuan brick tea. (**A**) Colony feature and microscopic feature of isolated *Aspergillus niger* PW-2: colony feature after 4 days at 25 °C on PDA (a,b), MEA (c,d) and CYA (g,h), and colony feature after 7 days at 25 °C on CDA (e,f); morphologic characteristics of conidiophores (i), septate hyphae asymmetrically branched (j) and conidia (k) of *Aspergillus niger* PW-2 under a light microscope. (**B**) Neighbor-joining phylogenetic tree based on the dataset covering the sequences of the ITS gene of isolates and reference strains.

The strain was isolated from Pingwu Fuzhuan brick tea, and the isolated strain was cultured on CDA at 25 °C for 7 days, and on PDA, CYA and MEA at 25 °C for 4 days. The morphological features of the strain were observed under a light microscope [14]. The ITS gene of the strain was amplified by PCR using primers ITS1 (5′-TCCGTAGGTGAACCTGCGG-3′) and ITS4 (5′-TCCTCCGCTTATTGATATGC-3′) at Sangon Biotech Co., Ltd. (Shanghai, China). Sequences with high similarity to the sequence of the ITS gene were obtained using BLAST (https://blast.ncbi.nlm.nih.gov/Blast.cgi, last accessed on 21 February 2022), and the phylogenetic tree was constructed using MEGA 6.0 software. The strain was identified to be *A. niger* PW-2 (CCTCC NO: M 2020618) (Figure 1).

### 2.3. Preparation of A. niger PW-2-Fermented Steamed Green Tea

The fermentation by *A. niger* PW-2 was performed as described previously with slight modifications [10,15]. In brief, steamed green tea leaves (720 g) were sterilized at 121 °C for 20 min. After cooling down to room temperature, the sterilized tea leaves were inoculated with 270 mL of *A. niger* PW-2 spore suspension at a concentration of 1.44 × 10^6^ CFU/mL and then fermented at 28 °C for 21 days. Fermented tea samples were collected after 0, 3, 6, 9, 12, 15, 18 and 21 days. Three grams of fermented tea samples was stored at −80 °C until subsequent proteomics analysis, and the remaining samples were dried at 50 °C for 24 h and then ground for use in metabolite analysis.

### 2.4. Taste Evaluation by Electronic Tongue

An electronic tongue system (TS-5000Z, INSENT, Atsugi, Japan) was used to determine the taste intensity of the infused tea. The sample preparation and electronic tongue analysis were conducted as described previously [16,17]. Six types of taste sensors, including AAE, CT0, CA0, C00, AE1 and GL1, were used to analyze umami taste, saltiness, sourness, bitterness, astringency and sweetness, respectively.

### 2.5. Analysis of Metabolites

#### 2.5.1. Aroma Composition Analysis by HS-SPME/GC-MS

Powdered tea samples (0.5 g) were infused in 5 mL of boiling water, and 20 μL of ethyl decanoate (20 μg/mL) was added to the infusion as the internal standard. Volatile component extraction was conducted according to the method reported previously using a solid-phase microextraction (SPME) fiber coated with DVB/CAR/PDMS (Supelco, Sigma Aldrich, Tokyo, Japan) in conjunction with gas chromatography mass spectroscopy (GC-MS) (GCMS-QP2010 SE, Shimadzu, Kyoto, Japan) [15]. The NIST MS data library and Kovats’ retention indices (RI) were used to identify volatile components. The volatile contents were expressed as mg ethyl decanoate/kg tea.

#### 2.5.2. Analysis of Free Amino Acids

Samples used for free amino acid analysis were prepared based on the method previously reported with some modifications [18]. Tea powder (0.25 g) was added to 10 mL of boiling water, and the mixture was stirred in a boiling water bath for 30 min using a magnetic stirrer (DF-101S, Shanghai Qiuzuo Scientific Instruments Co., Ltd., Shanghai, China); after that, it was cooled down to room temperature. After centrifugation at 10,000× *g* for 10 min, 800 μL of the supernatant was deproteinized by mixing with 200 μL of 10% sulfosalicylic acid overnight at 4 °C. The mixture was subsequently centrifuged at 15,000× *g* for 10 min, and the supernatant was filtered through a 0.22 μm nylon membrane. Finally, 20 μL of the obtained sample was loaded onto an A300 automatic amino analyzer (Membrapure GmbH, Berlin, Germany).

#### 2.5.3. Determination of Gallic Acid, Catechin and Alkaloid Contents

Gallic acid, catechin and alkaloid contents were determined following the method established in our laboratory [15]. In brief, tea samples (0.5 g) were extracted with 70% aqueous methanol (20 mL) by sonicating for 15 min, and the extracts were then filtered through a 0.22 μm nylon membrane. Then, the filtrates were diluted with 70% aqueous methanol at a ratio of 1:1 for HPLC analysis. HPLC analysis was carried out using a Thermo Ultimate 3000 HPLC system (Thermo, Waltham, MA, USA) equipped with an Ultimate 3000 diode array detector (detection wavelength, 280 nm) coupled to an Inertisil ODS-4 column (5 μm, 4.6 mm × 250 mm; GL-science Inc., Tokyo, Japan), the temperature of which was maintained at 30 °C. The flow rate was set at 0.8 mL/min, and the injection volume was 10 μL. Mobile phase A consisted of 0.1% formic acid in water, and mobile phase B consisted of methanol. The elution gradient was programmed as follows: 0–5 min, 5–22% B; 5–20 min, 22% B; 20–35 min, 22–24% B; 35–45 min, 24–25% B; 45–50 min, 25–40% B; and 50–60 min, 40–45% B.

#### 2.5.4. Determination of Color Difference among Tea Infusions, and Contents of Total Polyphenols, Flavonoids and Tea Pigments

The difference between the colors of different tea infusions was analyzed using a CM-5 spectrophotometer (Konica Minolta, Tokyo, Japan) as described previously [19]. The L*, a* and b* values represent lightness (100 to 0, white to black), redness (+red to −green) and yellowness (+yellow to −green), respectively. The Folin–Ciocalteu method [20] and aluminum trichloride colorimetric method [21] were used to determine the contents of total polyphenols (mg GA/g tea) and total flavonoids (mg rutin/g tea), respectively. Tea pigments (theaflavins, thearubingins, theabrownins) were determined following the method described previously [22].

### 2.6. Proteomics Analysis

The proteomics analysis of microbial proteins in tea leaves was carried out according to the method described previously with some modifications [10]. The microbial proteins were extracted with tris-saturated phenol and then precipitated with 0.1 M ammonium acetate in methanol. Then, trypsin was added at a trypsin/protein mass ratio of 1:50 to digest the proteins overnight at 37 °C, and the trypsin-digested peptide was desalted and then quantified. Liquid chromatography-tandem mass spectrometry (LC-MS/MS) was employed to analyze the peptide extracts. The methods are described in detail in Supplementary methods, Supplementary Material. The MS/MS data of the proteins were searched against the NCBI database (https://www.ncbi.nlm.nih.gov/, last accessed on 21 February 2022). The functions of the identified proteins were annotated according to the GO, KEGG and CAZy databases.

### 2.7. Data Analysis

Significant differences were determined by SPSS software (v22.0, SPSS inc., Chicago, IL, USA) using Student’s *t*-test and Duncan’s multiple range test. Partial least squares discriminant analysis (PLS-DA) and orthogonal partial least squares discriminate analysis (OPLS-DA) were performed using SIMCA-P (v14.1, Umetrics AB, Umea, Sweden). R (v4.0.4) was used to generate heat maps. The metabolic pathways of differential metabolites were identified according to the KEGG pathways.

## 3. Results

### 3.1. Changes in Volatile Compounds during Fermentation by A. niger PW-2

**Figure 2:**
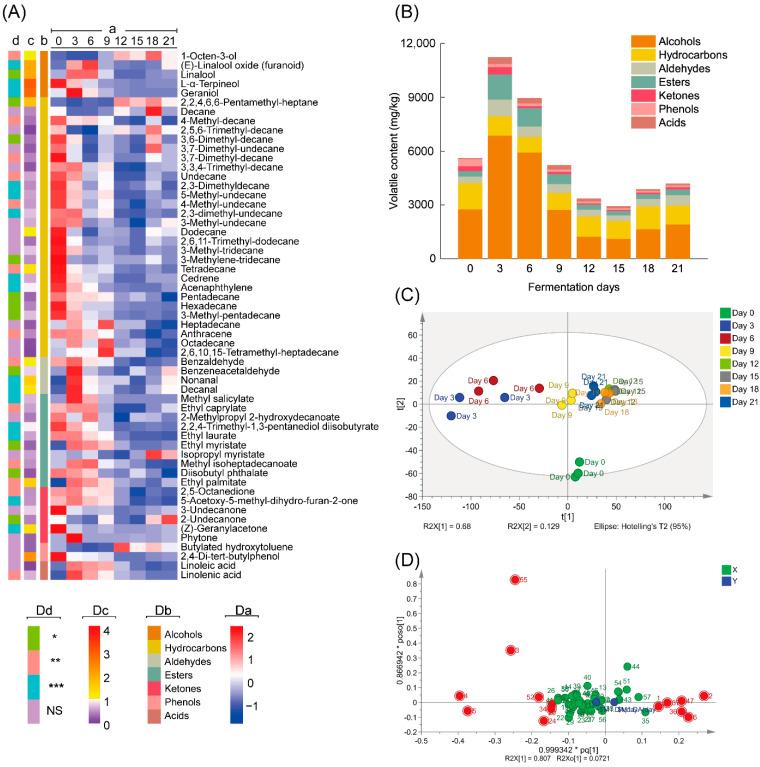
Information of the volatile compounds for the tea samples fermented by *Aspergillus niger* PW-2. (**A**) Heatmap representing the semiquantitative results (a), chemical classes (b), VIP values (c) and *p* values (d) of all volatile compounds (NS, *p* > 0.05; *, 0.01 < *p* < 0.05; **, 0.001 < *p* < 0.01; ***, *p* < 0.001). (**B**) Volatile contents of different classes in samples during fermentation. (**C**) Score scatter plot of PLS-DA analysis based on volatile compounds of all samples. (**D**) Loading scatter plot of OPLS-DA analysis based on volatile compounds of samples on Day 0 and Day 21.

After screening based on their MS data and retention indices, 57 volatile compounds, including 5 alcohols, 28 hydrocarbons, 4 aldehydes, 10 esters, 6 ketones, 2 phenols and 2 acids, were identified in all samples during the whole fermentation process (Figure 2A, Table S1).

As shown in Figure 2B, during the fermentation process, the total contents of volatile compounds initially increased from 5599.97 mg/kg (Day 0) to 11,218.83 mg/kg (Day 3) and declined to 4182.77 mg/kg at the end of fermentation (Day 21). The change trends of alcohol, hydrocarbon, aldehyde, ester, ketone and acid contents were consistent with the trends of total volatile contents, except for volatile phenol contents, which declined during the whole fermentation process. Alcohols, which accounted for 37.17–66.28% of the total volatile compounds, were the most abundant class of volatile compounds in teas during all fermentation stages, followed by hydrocarbons, which accounted for 9.77–33.88% of the total volatile compounds. Linalool was the most abundant volatile compound. PLS-DA score plots (Figure 2C) showed that samples on Day 12, Day 15 and Day 18 were clustered together and were distinguishable from samples at other fermentation stages. Principal components 1 and 2 explained 68% and 12.9% of the total variance, respectively. The loading plots (Figure 2D) and VIP values (Figure 2A, Table S1) obtained from OPLS-DA analysis of samples on Day 0 and Day 21 were used to identify the differential volatiles. The heatmap representing the volatile data and information of classes, VIP values and *p* values are illustrated in Figure 2A, and the detailed data are shown in Table S1. Volatile compounds with VIP > 1 and *p* < 0.05 (Figure 2A, Table S1) were considered differential compounds, and these compounds included 1-octen-3-ol, linalool, geraniol, *L*-*α*-terpineol, (*E*)-linalool oxide (furanoid), 2,2,4,6,6-pentamethyl-heptane, decanal, tetradecane, benzaldehyde, nonanal, ethyl palmitate, (*Z*)-geranylacetone, 2,4-di-tert-butylphenol and dodecane. Among these differential volatile compounds, the contents of 1-octen-3-ol, (*E*)-linalool oxide, 2,2,4,6,6-pentamethyl-heptane, nonanal, decanal and ethyl palmitate increased (*p* < 0.05) after the 21-day fermentation (Figure 2A). By contrast, the contents of linalool, geraniol, *L*-*α*-terpineol, tetradecane, benzaldehyde and (*Z*)-geranylacetone significantly decreased (*p* < 0.05) after the same fermentation period.

### 3.2. Changes in Chemical Compounds during Fermentation by A. niger PW-2

**Figure 3:**
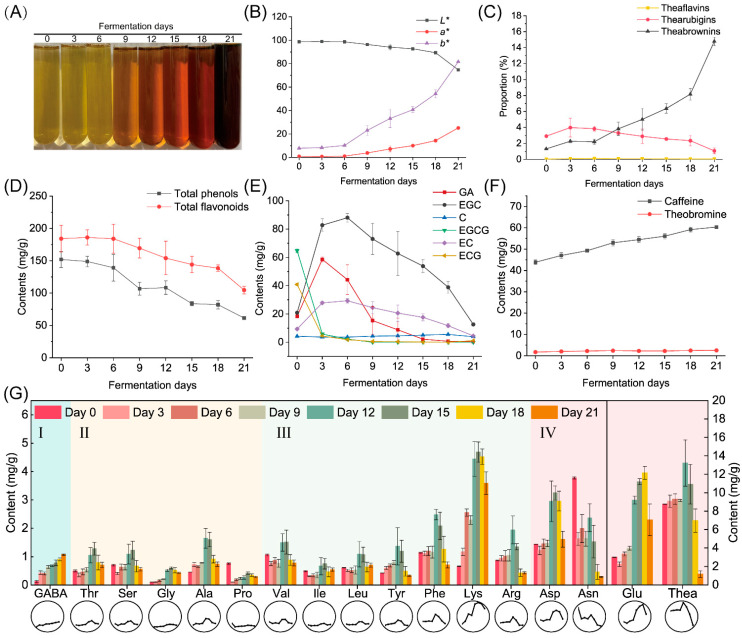
Physicochemical composition changes in samples fermented by *Aspergillus niger* PW-2. (**A**) Tea infusions. (**B**) Color difference among tea infusions. (**C**) Proportion of tea pigments including theaflavins, thearubigins and theabrownins. (**D**) Contents of total phenols and total flavonoids. (**E**) Contents of GA and catechins. (**F**) Contents of caffeine and theobromine. (**G**) Contents of flavor free amino acids ((I) astringency; (II) sweetness; (III) bitterness; (IV) umami), where the line chart on the bottom displays the trend visually. GA, gallic acid; GABA, γ-aminobutyric acid; Thr, threonine; Ser, serine; Gly, glycine; Ala, alanine; Pro, proline; Val, valine; Ile, isoleucine; Leu, leucine; Tyr, tyrosine; Phe, phenylalanine; Lys, lysine; Arg, arginine; Asp, aspartate; Asn, asparagine; Glu, glutamate; Thea, theanine.

Microbial fermentation can influence the color of tea infusions, as shown in Figure 3A. The tea infusion color gradually changed from yellowish-greenish to reddish-brownish. The changes in color values L*, a* and b* (Figure 3B, Table S2) were also consistent with the changes in tea infusion color: the L* value decreased from 98.67 to 74.72, the a* value increased from 0.96 to 25.2 and the b* value increased from 7.78 to 81.63. The changes in tea pigment concentrations are shown in Figure 3C and Table S2. During fermentation by *A. niger* PW-2, the theabrownins content was found to steadily increase from 1.32% to 14.79%. Other pigments such as theaflavins and thearubigins were also detected in tea samples. During the entire fermentation period, the contents of theaflavins remained at a low level (<0.1%), whereas those of thearubigins ranged between 1% and 3%.

Phenols are the major contributors to the taste and health benefits of teas. During fermentation with *A. niger* PW-2, the contents of total phenols and total flavonoids significantly decreased by 59.63% and 43.15%, respectively (Figure 3D, Table S2). The contents of gallate catechins EGCG and ECG declined from 64.75 and 40.81 mg/g, respectively, on Day 0 to 0 and 1.15 mg/g, respectively, on Day 21 (Figure 3E, Table S2). In addition, the contents of non-gallate catechins EGC and EC increased from 20.95 and 9.38 mg/g, respectively, on Day 0 to 88.06 and 29.32 mg/g, respectively, on Day 6 and then gradually decreased to 12.65 and 4.48 mg/g, respectively, on Day 21. Similarly, the content of gallic acid increased from 18.38 mg/g on Day 0 to 58.62 mg/g on Day 3 and then decreased to 0.41 mg/g on Day 21.

The main alkaloids found in teas include caffeine, theobromine and theophylline. As shown in Figure 3F and Table S2, theophylline was not detected during the whole fermentation period. Theobromine contents were stable at a low level, while caffeine contents steadily increased from 43.86 to 60.35 mg/g.

A total of 17 amino acids were detected in the tea samples. The contents of total amino acids increased from 22.6 to 39.33 mg/g in the first 15 days of fermentation and then finally decreased to 16.71 mg/g. As shown in Figure 3G and Table S2, the contents of most amino acids reached their peak values at the mid-fermentation stage (Day 12 to Day 15) and decreased thereafter. In particular, the content of γ-aminobutyric acid (GABA) increased continuously during the whole fermentation period. The contents of sweet amino acids including threonine, serine, glycine, alanine and proline, with a range of 0.29–0.73 mg/g in the finished sample, were relatively low compared to those of bitter and umami amino acids. During fermentation, the contents of threonine, glycine and alanine increased by 42.29%, 3.3-fold and 62.30%, respectively, whereas those of serine and proline decreased by 20.61% and 62.15%, respectively. Among all bitter amino acids, including valine, isoleucine, leucine, tyrosine, phenylalanine, lysine and arginine, lysine was the most abundant bitter amino acid in the finished sample; its content significantly increased by 4.36-fold during fermentation. In addition, the contents of isoleucine and leucine slightly increased by 11.09% and 14.26%, respectively, whereas those of valine, tyrosine, phenylalanine and arginine decreased by 26.96%, 22.18%, 37.58% and 50.02%, respectively. Umami amino acids including aspartate, asparagine, glutamate and theanine were the most abundant group of amino acids detected in the finished sample, and the content of glutamate was the highest among all amino acids. After fermentation for 21 days, the content of aspartate increased from 1.44 to 1.51 mg/g, whereas that of glutamate increased from 3.00 to 7.07 mg/g. The contents of asparagine significantly dropped from 3.78 to 1.87 mg/g (51.68%), while those of theanine dropped from 8.77 to 1.17 mg/g (86.66%).

**Figure 4:**
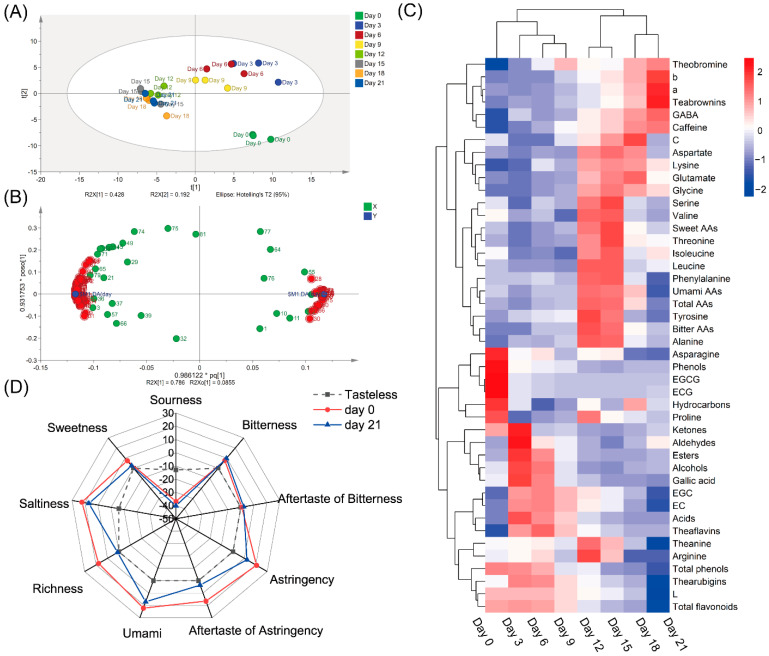
The analysis of taste characteristics and the dynamics changes of metabolites in tea samples during fermentation. (**A**) Score scatter plot of PLS-DA analysis based on all metabolites detected in all fermented samples. (**B**) Loading scatter plot of OPLA-DA analysis based on all metabolites detected in samples on Day 0 and Day 21. (**C**) Results of heatmap analysis. (**D**) The taste scores of samples on Day 0 and Day 21. GABA, γ-aminobutyric acid; AAs, amino acids.

As shown in Figure 4A, the PLS-DA analysis of 90 metabolites indicated that the fermented samples on Day 0, Day 3, Day 6 and Day 9 were clearly separated, while other samples from Day 12 to Day 21 were clustered together. Based on their VIP values and *p* values (Table S3), 60 metabolites (VIP > 1, *p* < 0.05) between samples on Day 0 and Day 21 were differential metabolites (Figure 4B). The heatmap (Figure 4C) generated based on metabolites in tea samples fermented by *A. niger* PW-2 showed that the metabolites could be divided into three groups: Group I, the level of the chemical increased in the final sample on Day 21; Group II, the level of the chemical increased in the samples on Days 12–18 and then decreased on Day 21; Group Ⅲ, the level of the chemical was high during the early fermentation stage (Days 0–9) but was low during the subsequent fermentation stage.

### 3.3. Changes in Tea Taste Quality after Fermentation by A. niger PW-2

The electronic tongue system equipped with an artificial lipid membrane capable of consistently responding to taste similar to the human tongue was used to measure the taste intensity of the tea infusion [16]. Because the scores of sourness were lower than those of the tasteless point, the taste of sourness in tea samples was not measured before and after fermentation (Figure 4D). The sweetness on Day 0 was at a low level and was reduced to nearly the tasteless point on Day 21. The intensities of bitterness and bitter aftertaste on Day 21 were slightly higher compared to those on Day 0, all of which had relatively low levels. The intensities of umami taste on Day 21 were lower than those on Day 0, and the richness (the persistence of umami) changed significantly after fermentation. It is worth noting that the intensities of astringency and astringent aftertaste also reduced significantly.

### 3.4. Proteomic Analysis

A total of 1994 proteins were identified in all tea samples during fermentation by *A. niger* PW-2 (Table S4). The changes in the abundance of all proteins are illustrated by a heatmap displayed in Figure S1. The GO classes that were highly represented included the cellular process and metabolic process of the biological process; the cellular anatomical entity of the cellular component; and the catalytic activity and binding of the molecular function (Figure S2A and Table S5). The KEGG pathways that were enriched included “carbohydrate metabolism” (329), “amino acid metabolism” (261), “energy metabolism” (127), “lipid metabolism” (97), “metabolism of other amino acids” (95), “metabolism of cofactors and vitamins” (89), “nucleotide metabolism” (59), “glycan biosynthesis and metabolism” (26) and “metabolism of terpenoids and polyketides” (17) (Figure S2B and Table S5). The CAZymes that are responsible for the synthesis of all carbohydrates are glycosyltransferases (GTs), and those involved in the degradation include carbohydrate esterases (CEs), glycoside hydrolases (GHs), auxiliary activities (AAs) and polysaccharide lyases (PLs); carbohydrate binding modules (CBMs) are responsible for recognition. The CAZymes identified in this study could be divided into GHs (103), AAs (23), CBMs (15), CEs (25), GTs (14) and PLs (6) (Table S5).

**Figure 5:**
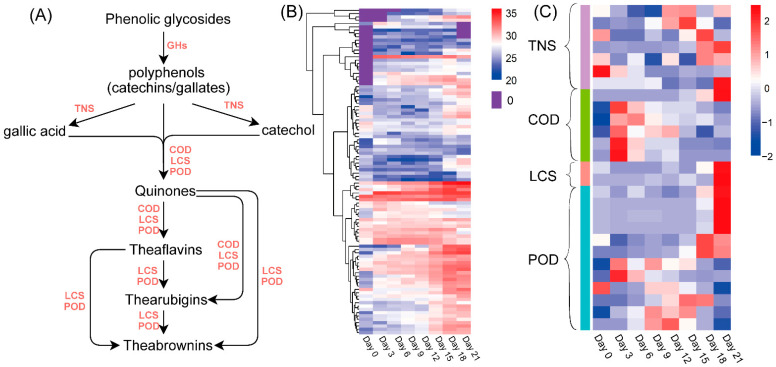
The hypothetical metabolic pathway of tea phenols (**A**), and the abundance heatmap of related enzymes: heatmap of GHs (**B**), and heatmap of TNS, COD, LCS and POD (**C**). GHs, glycoside hydrolases; TNS, tannase; COD, catechol oxidase; LCS, laccase; POD, peroxidase.

The proposed metabolic pathway of tea phenols is shown in Figure 5A, and the abundances of the related proteins are shown as heatmaps in Figure 5B,C. The heatmap of GHs (Figure 5B and Table S6) showed that the abundance of most GHs increased during the whole fermentation period and was the highest at the late fermentation stage (Days 18–21). Seven proteins in the tannase and feruloyl esterase family (TNS) were identified by manual checking (Figure 5B and Table S7). Most of these proteins had higher levels at the late fermentation stage (Days 15–21). Tea phenols including catechins, gallates, gallic acids and catechol were oxidized to quinones by catechol oxidase (COD), peroxidase (POD) or laccase (LCS) during fermentation. As shown in Figure 5A, as the fermentation degree was increased, more quinones were converted to theaflavins, thearubigins and theabrownins by COD, POD and LCS [23]. The levels of most CODs obviously increased on Day 3 of fermentation and then decreased. The levels of some proteins in the COD and POD groups also increased during the mid-fermentation stage (Days 3–18), while those of other proteins in the COD, LCS and POD groups reached their maximum levels at the end of fermentation (Day 21) (Figure 5B and Table S7).

**Figure 6:**
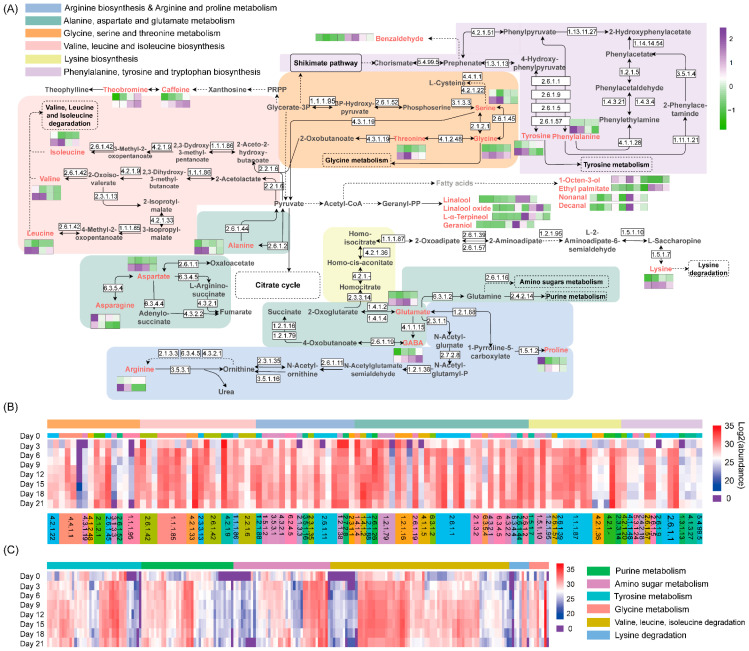
The map of the metabolic pathway based on differential metabolites (**A**), and the heatmap analysis of enzymes related to the biosynthesis and metabolism of metabolites (**B**,**C**). GABA, γ-aminobutyric acid.

To better understand the dynamic changes in metabolites during fermentation by *A. niger* PW-2, the proposed metabolic pathway analyzed based on the KEGG pathway was annotated with the determined metabolites and enzymes (Figure 6A). The changes in the contents of metabolites including amino acids, differential volatile compounds and alkaloids are also included in Figure 6A. The profiles of enzymes involved in amino acid synthesis and degradation are shown by a heatmap in Figure 6B,C, respectively, and the detailed information is shown in Table S7.

## 4. Discussion

In the present study, the levels of most metabolites first increased and then decreased. One possible reason for this observation is that during the fermentation by *A. niger* PW-2, enzymes such as cellulases, α/β-glucosidases and pectin lyase, which could degrade plant polysaccharides, were secreted, resulting in the lysis of the cell walls of tea leaves, causing the release of more metabolites. Oligosaccharide produced from the degradation of polysaccharides might be responsible for the mellow taste of fermented tea [10]. Additionally, monosaccharides might enter into glycolysis, acting as a flux of the carbon supply for secondary metabolisms, such as purine and amino acid metabolisms [24].

Compared to pan-fired green tea, steam-treated green tea has lower levels of geraniol, and linalool and its derivatives [13]. Among all differential volatiles, linalool, geraniol, *L*-*α*-terpineol and (*E*)-linalool oxide (furanoid), which give a floral aroma, were generated from the aroma precursors by microbial GHs (Table S6) [6]. 1-Octen-3-ol with an intense, persistent mushroom-like odor was derived from the oxidative degradation of linoleic acid. Nonanal (fatty, citrus-like, green), decanal (soap, orange peel, tallow) and hexadecanoic acid ethyl ester (cheese-like) originated from the oxidative degradation or esterification of fatty acids, while benzaldehyde (fragrant, sweet and almond aroma) originated from the oxidation of amino acids (Figure 6A) [25,26]. Alcohol dehydrogenases and aldehyde dehydrogenases identified in tea samples fermented by *A. niger* PW-2 (Table S4) might be involved in the metabolism of aldehydes. The detected differential volatile hydrocarbons included dodecane, tetradecane and 2,2,4,6,6-pentamethyl-heptane. According to their numbers, hydrocarbons were the most abundant compounds; however, they contribute little to the aroma because they provide only a few types of odors. Generally, saturated hydrocarbons do not contribute to the aroma of tea, while unsaturated hydrocarbons play an important role in contributing to the flavor of tea [27].

Phenols, including phenolic glycosides, catechins and gallic acid, are the main source of astringency in teas [28]. As a previous study reported [4], the astringent scores of theabrownins at different concentrations were the same as the tasteless scores. The decrease in catechin and gallic acid contents and the increase in theabrownin contents might lead to the reduction in astringency and astringent aftertaste (Figure 4C,D) [28]. As illustrated in the proposed metabolic pathway of tea phenols (Figure 5A), the hydrolysis of the glycosidic bonds in phenolic glycosides by GHs produces phenolic aglycone [29]. Some phenols such as gallates can be hydrolyzed by tannases, by which the ester linkages are broken down to release small molecular phenols (e.g., catechol) and phenolic acids (e.g., gallic acid) (Figure 5A) [30]. As shown in Figure 3E, galloyl catechins EGCG and ECG were steadily degraded; their contents were reduced to nearly zero on Day 9. The contents of gallic acid and non-galloyl catechins (EGC and EC) increased during the first three or six days and then decreased until the end of fermentation. It is possible that galloyl catechins were degraded to non-galloyl catechins and gallic acid by the hydrolysis led by extracellular enzymes during the early fermentation stage, and non-galloyl catechins and gallic acid were further degraded to other simpler compounds such as pyrogallol, catechol and phenolic acids [1]. The level of metallo-dependent hydrolase (Figure 5C, Table S7) showed a decreasing trend in a similar fashion to that of galloylated catechins (EGCG and ECG), which may be related to the increase in gallic acids and non-galloylated catechins (EGC and EC) during the first three days of fermentation. The levels of most CODs (Figure 5C) obviously increased during the first 3 days of fermentation and then decreased, similar to the same trends of gallic acid, EGC and EC contents. Proteins in the COD and POD groups, of which the levels increased during the mid-fermentation stage, may play a role in the increase in theabrownin contents from Day 3 to Day 18. In addition, proteins in the COD, LCS and POD groups, of which the levels obviously increased at the end of fermentation (Day 21), may be related to the increase in theabrownins from Day 18 to Day 21 (Figure 5C, Table S7). The characteristic reddish-brown color of the fermented tea infusion was due to the presence of theabrownins (Figure 3A–C), which are a type of macromolecular pigment that consists of phenolic substances, alkaloids, polysaccharides, proteins and amino acids. Theabrownins are bioactive substances responsible for the hypocholesterolemic and hypolipidemic effects of dark teas [1,3]. The content of theabrownins in tea samples fermented for 21 days presented in this study was higher compared to that in the data summarized by a previous study [2].

Caffeine is the key contributor to the bitter taste of tea infusions [28]. Caffeine contents increased steadily during fermentation (Figure 3F), which is consistent with previous results [15,31]; however, this is contrary to the results reported by some studies [4,32]. The increase in caffeine may be the reason for the increase in the bitter taste after fermentation (Figure 4D). Caffeine cannot be easily degraded due to its stable cyclic structure [11]. Caffeine is a precursor of theophylline synthesis, which can be converted from xanthosine and interconverted to theobromine [33]. Theophylline and enzymes related to caffeine synthesis were not detected in our fermented tea samples, and the level of theobromine was low and nearly unchanged (Figure 3F). We hypothesized that the increase in the caffeine content may be related to the moist heat condition, the enzymes involved in caffeine biosynthesis not annotated in our study or not included in the KEGG database and the greater release of chemicals due to the degradation of plant polysaccharides. Moreover, the accumulated caffeine was not degraded due to the lack of a caffeine degradation pathway in *A. niger* PW-2.

Free amino acids have been considered as the key contributors to the aroma and taste of tea. The contents of total amino acids during the fermentation process by *A. niger* PW-2 first increased and then decreased (Table S2). The levels of free amino acids can contribute to the degree of biosynthesis or degradation of amino acids and proteins [34]. The abundance of enzymes involved in the biosynthesis of amino acids (Figure S3) remained stable during the whole fermentation, or it increased on Day 3 and then remained stable thereafter. This indicates that the increase in amino acid contents was not due to the enhancement of amino acid biosynthesis. The expressions of most enzymes involved in the protein biosynthesis pathways (Figure S3), such as ribosome and aminoacyl-tRNA biosynthesis, were obviously upregulated on Day 3, which was consistent with the decrease in the contents of some amino acids during the same period. From the fermentation stage of Day 12, the abundance of enzymes involved in the protein biosynthesis pathway was at a lower level. The expressions of enzymes related to protein degradation, including proteasome and ubiquitin-mediated proteolysis (Figure S3), were upregulated on Day 9 and Day 12, and this was somewhat in correspondence with the trend of the amino acid content. In general, with the effect of *A. niger* PW-2, the biosynthesis and degradation of proteins might be the main reason for the decrease and increase in amino acid contents.

The contents of sweet-taste amino acids were slightly increased (*p* > 0.05) in samples on Day 0 (2.51 mg/g) and Day 21 (2.72 mg/g) (Figure S4). This indicates that sweet-taste amino acids might not be the main contributor to the sweet taste of tea; the sweet taste might be derived from the degradation of carbohydrates. The level of bitter-taste amino acids increased during the whole fermentation by 34.63%, and that of umami-taste amino acids decreased by 40.27%, which was in accordance with the change trend of the taste score (Figure S4). GABA is a non-protein amino acid with a mouth-drying and velvety-like astringent taste that can be catalyzed by glutamate decarboxylase 1 (EC: 4.1.1.15) and 4-aminobutyrate aminotransferase (EC: 2.6.1.19). We found that 4-aminobutyrate aminotransferase might be the main enzyme related to the generation of GABA, as the abundance of both had the same change trend (Figure 5A,B). Theanine may be degraded into glutamate and ethylamine by enzymes or moist heat conditions, which generates catechins and GABA [35].

## 5. Conclusions

In summary, this study provided an in-depth understanding of the enzymes secreted by *A. niger* PW-2, revealing the bioconversion and improvement in the quality of steamed green tea fermented by *A. niger* PW-2 using proteomic analysis. The *A. niger* PW-2 fermented steamed green tea had lower contents of linalool, geraniol and *L*-*α*-terpineol which contribute to the “green” aroma, while it had higher contents of 1-octen-3-ol, nonanal, decanal and ethyl palmitate which contribute to mushroom, citrus-like and cheese-like odors. After fermentation, the astringent taste was significantly weakened, mainly due to the decrease in the contents of polyphenols, such as catechins. The contents of theabrownins increased significantly, causing the tea infusion to have a reddish-brown color. *Aspergillus niger* PW-2 was found to secret enzymes that can degrade plant polysaccharides and phenolic compounds, as well as those that can produce theabrownins. The strain also secreted enzymes involved in the biosynthesis and degradation of proteins, causing the amino acid contents to change. This study advances our understanding of the mechanism of *A. niger* PW-2 that influences the conversion of chemical compounds and the formation of unique sensory characteristics of fermented steamed green tea.

## Data Availability

Not applicable.

## Supplementary Materials

The following supporting information can be downloaded at: https://www.mdpi.com/article/10.3390/foods11060865/s1, Figure S1: Heatmap analysis based on all proteins detected in all fermented samples; Figure S2: GO annotation classification (A) and KEGG pathway classification of all proteins detected in all fermented samples; Figure S3: Heatmap analyses of enzymes related to the pathways of the biosynthesis of amino acids (A), ribosome (B), aminoacyl-tRNA biosynthesis (C), proteasome (D) and ubiquitin mediated proteolysis (E); Figure S4: Analysis of amino acids summed by sweet, bitter, umami and astringent taste; Supplementary methods: The methods of proteomics analysis; Table S1: Contents of volatile compounds identified in fermented teas; Table S2: Physicochemical properties of fermented teas; Table S3: VIP values and *p* values of all chemical factors of analysis on samples of Day 0 and Day 21; Table S4: All proteins identified in fermented tea samples; Table S5: Annotations of proteins by GO, KEGG and CAZymes; Table S6: Enzymes involved in glycoside hydrolases (GHs); Table S7: Enzymes involved in TNS, COD, LCS and POD; Table S8: Enzymes related to amino acids metabolism.
