# Supplementary material for: Delving into the Biotransformation Characteristics and Mechanism of Steamed Green Tea Fermented by Aspergillus niger PW-2 Based on Metabolomic and Proteomic Approaches
Source: Foods. 2022 Mar 18;11(6):865. doi: 10.3390/foods11060865 (PMC8951510; doi:10.3390/foods11060865)
Supplement: Supplementary file 1 [file foods-11-00865-s001.zip › Supplementary Figures.pdf]

Supplementary Figures

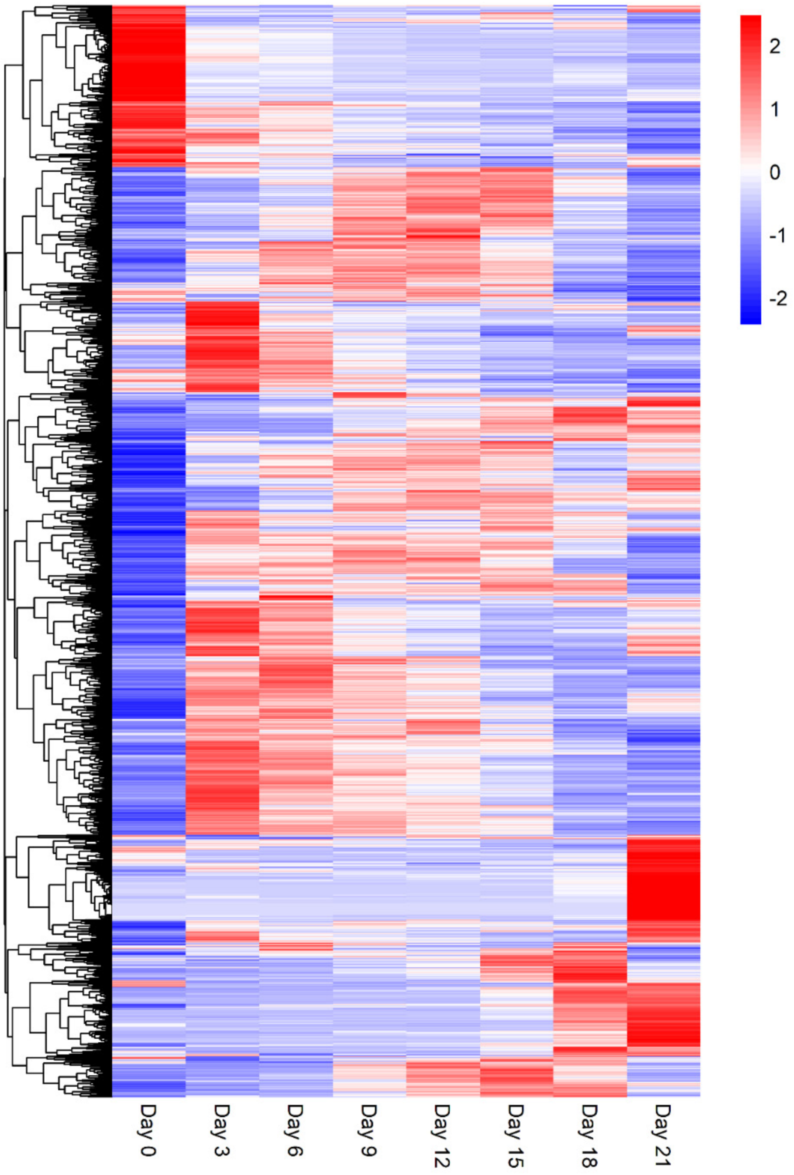

Figure S1. Heatmap analysis based on all proteins detected in all fermented samples.

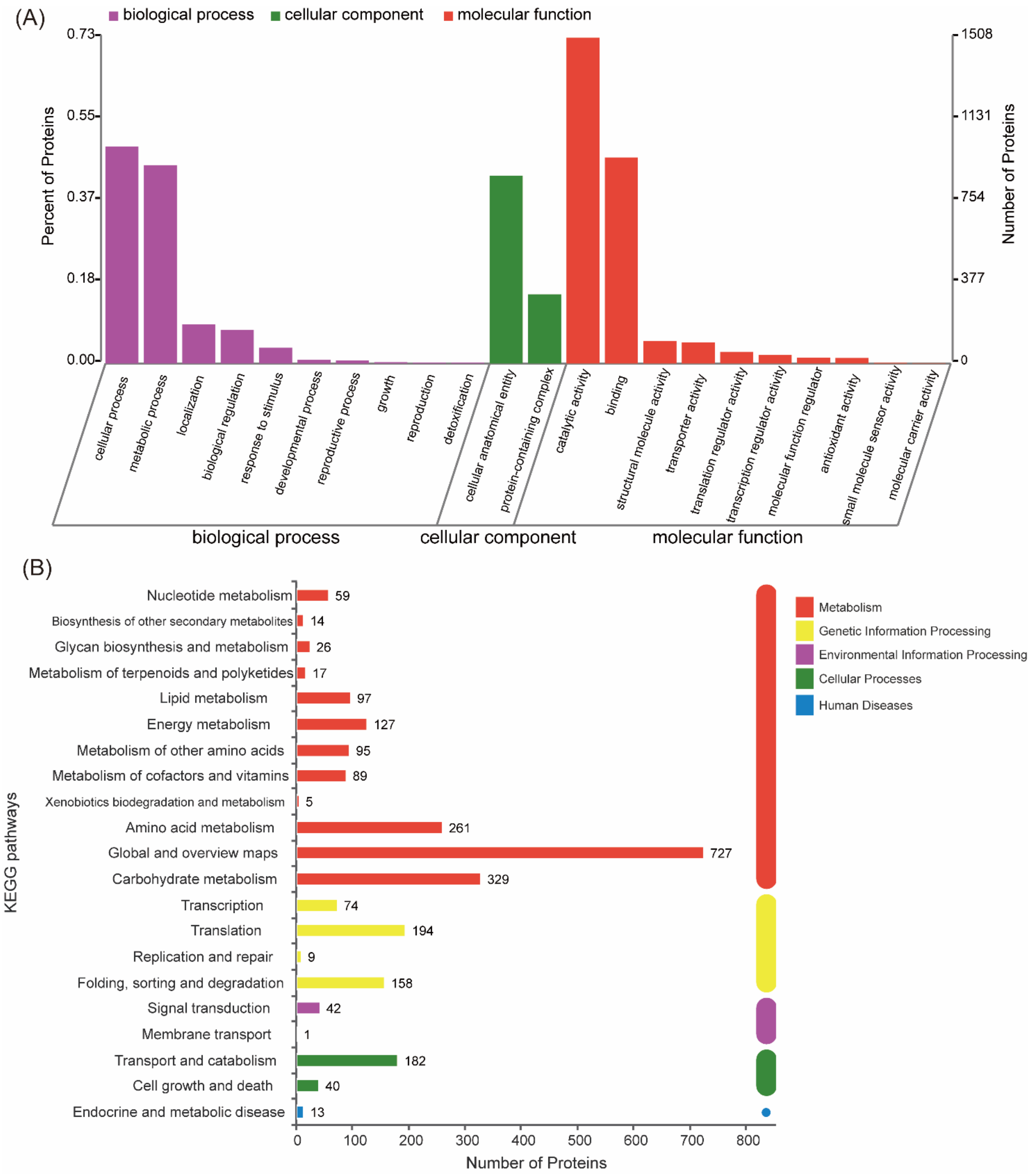

Figure S2. GO annotation classification (A) and KEGG pathway classification of all proteins detected in all fermented samples.

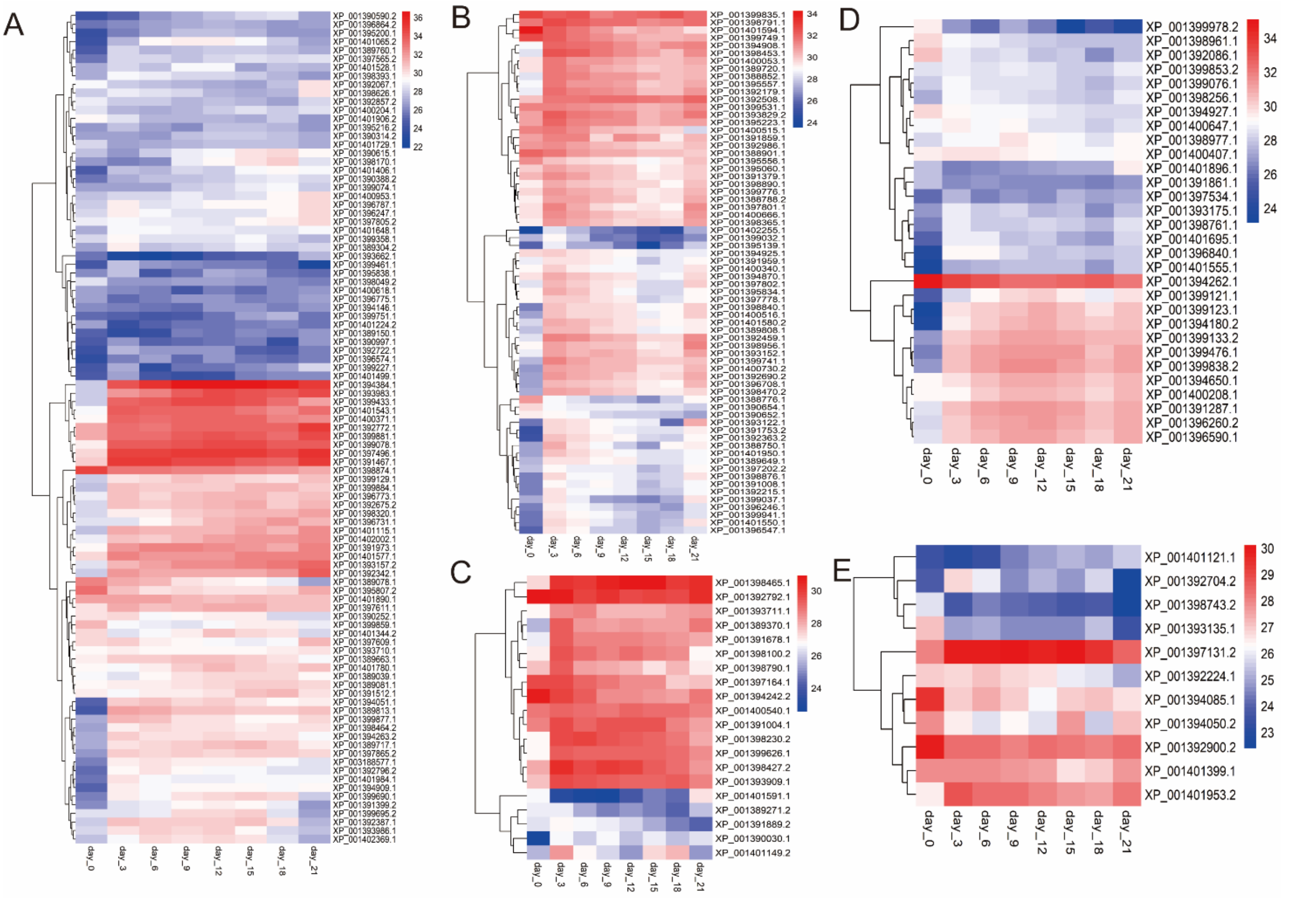

Figure S3. Heatmap analyses of enzymes related to the pathways of the biosynthesis of amino acids (A), ribosome (B), aminoacyl-tRNA biosynthesis (C), proteasome (D) and ubiquitin mediated proteolysis (E).

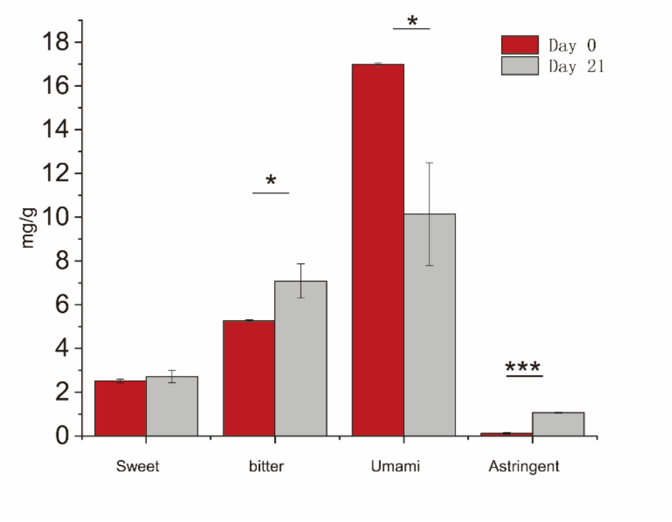

Figure S4. Analysis of amino acids summed by sweet, bitter, umami and astringent taste.
