# Supplementary material for: Delving into the Biotransformation Characteristics and Mechanism of Steamed Green Tea Fermented by Aspergillus niger PW-2 Based on Metabolomic and Proteomic Approaches
Source: Foods. 2022 Mar 18;11(6):865. doi: 10.3390/foods11060865 (PMC8951510; doi:10.3390/foods11060865)
Supplement: Supplementary file 1 [file foods-11-00865-s001.zip › Supplementary Methods.pdf]

Supplementary methods

Proteomics analysis. Appropriate tea samples were suspended in 1% PVPP and BPP solution. The solution was centrifuged at 12000 g for 20 min at 4°C, and supernatants were collected. The equal volume of Tris-saturated phenol was added and vortexed for 10 min at 4°C. The solution was centrifuged at 12000 g for 20 min at 4°C and the phenol phase were collected. The equal volume of BPP was added and vortexed for 10 min at 4°C, then centrifuged at 12000 g for 20 min at 4°C. Extracted proteins were precipitated from the collected phenol phase by adding five volumes of pre-cooled 0.1M ammonium acetate in methanol, incubating overnight at -20°C and then collecting by centrifugation at 12000 g for 20 min at 4°C. The collected protein pellets were washed twice with cold 90% acetone. The remain pellets were air-dried and solubilized with lysis buffer (1% SDS, 8 M urea, cocktail), followed by sonicating on ice for 2 min. After centrifugation at 12000 g for 30 min at 4°C, the supernatants were collected for later digestion.

For digestion, TEAB was added to a final concentration of 100 mM, then TCEP was added to a final concentration of 100 mM and placed at 37°C for 60 min. Iodoacetamide was added to a final concentration of 40 mM and reacted at room temperature in the dark for 40 min. Six volumes of cold acetone were added and precipitated for 4 h at -20 °C, then centrifugated at 10000 g for 20 min at 4°C. The collected precipitated proteins were resuspended with 100 μL of 100 mM TEAB, and trypsin was added at a 1:50 trypsin-to-protein mass ratio for digestion overnight at

37°C. the trypsin-digested peptide was desalted and quantified with kit.

Liquid chromatography-tandem mass spectrometry (LC-MS/MS) analysis of each replicate of peptide extracts was performed using an Easy-nLC 1200 coupled to a Q-Exactive HF-X mass spectrometer (Thermo Fisher Scientific, Bremen, Germany). Peptides (0.25 μg/μL) were loaded onto a C18 column (3 μm, 75 μm × 250 mm, Thermo ,USA). The mobile phase consisted of (A) 2% acetonitrile containing 0.1% formic acid (*v*/*v*) and (B) 80% acetonitrile containing 0.1% formic acid (*v*/*v*). The LC gradient was set as follows: 0-53 min, 5-23% B; 53-65 min, 23-29% B; 65-73 min, 29-38% B; 73-74 min, 38-48% B; 74-75 min, 48-100% B; 75-90 min, 100% B. The flow rate was 300 nL/min. MS data were acquired in a data-dependent mode. The spray voltage was set to 2.1 kV, the heated capillary temperature was 280 °C. Survey full scan MS spectra (from *m*/*z* 350 to 1500) were acquired with a resolution of 60,000 and an AGC (Automatic Gain Control) target value of 3×10^6 ions. The 20 most intense precursors were selected for higher-energy collisional dissociation (HCD) MS/MS (resolution, 15,000; AGC target at 1e5; maximum fill time, 50 ms; fixed first mass, 100 m/z; minimum AGC target, 8e3; intensity threshold, 1.6e5; exclusion duration, 18 s).

Raw data were processed using Thermo Proteome Discoverer software version 2.2 (Thermo Fisher Scientific, Bremen, Germany) with the default settings. The MS/MS data were queried against the NCBI database (https://www.ncbi.nlm.nih.gov/) with the following search parameters: carbamidomethylation of cysteine as the fixed modification, oxidation of methionine and deamidation of glutamine and asparagine

as variable modifications, a maximum of two missed cleavages, a precursor ion mass tolerance of 10 ppm and an MS/MS tolerance of 0.05 Da. Decoy database searches were performed with a false discovery rate (FDR) cutoff of 1%.
